# Study of enzymatic properties of phenol oxidase from nitrogen-fixing *Azotobacter chroococcum*

**DOI:** 10.1186/2191-0855-1-14

**Published:** 2011-06-24

**Authors:** Susanne Herter, Marlen Schmidt, Mark L Thompson, Annett Mikolasch, Frieder Schauer

**Affiliations:** 1 Institute of Microbiology, Department of Applied Microbiology, University of Greifswald, Friedrich-Ludwig-Jahn-Str. 15a, 17487, Greifswald, Germany; 2 Institute of Biochemistry, Department of Biotechnology & Enzyme Catalysis, University of Greifswald, Felix-Hausdorff Str. 4, 17487, Greifswald, Germany

**Keywords:** Bacterial phenol oxidase, laccase, tyrosinase, *Pycnoporus cinnabarinus*, *Agaricus bisporus*, nitrogen fixation, cysts, melanin, oxygen protection

## Abstract

*Azotobacter chroococcum *is a widespread free-living soil bacterium within the genus of *Azotobacter *known for assimilation of atmospheric nitrogen and subsequent conversion into nitrogenous compounds, which henceforth enrich the nitrogen content of soils. *A. chroococcum *SBUG 1484, isolated from composted earth, exhibits phenol oxidase (PO) activity when growing under nitrogen-fixing conditions. In the present study we provide incipient analysis of the crude PO activity expressed by *A. chroococcum *SBUG 1484 within comparative analysis to fungal crude PO from the white-rot fungus *Pycnoporus cinnabarinus *SBUG-M 1044 and tyrosinase (PPO) from the mushroom *Agaricus bisporus *in an attempt to reveal desirable properties for exploitation with future recombinant expression of this enzyme. Catalytic activity increased with pre-incubation at 35°C; however 70% of activity remained after pre-treatment at 50°C. Native *A. chroococcum *crude PO exhibited not only strong preference for 2,6-dimethoxyphenol, but also towards related methoxy-activated substrates as well as substituted *ortho*-benzenediols from over 40 substrates tested. Presence of CuSO_4 _enhanced crude phenol oxidase activity up to 30%, whereas NaN_3 _(0.1 mM) was identified as the most inhibiting substance of all inhibitors tested. Lowest inhibition of crude PO activity occurred after 60 minutes of incubation in presence of 15% methanol and ethanol with 63% and 77% remaining activities respectively, and presence of DMSO even led to increasing oxidizing activities. Substrate scope and inhibitor spectrum strongly differentiated *A. chroococcum *PO activity comprised in crude extracts from those of PPO and confirmed distinct similarities to fungal PO.

## Introduction

Laccases (benzenediol:oxygen oxidoreductases, EC 1.10.3.2.) and related copper-containing proteins have been widely described in a considerable number of eukaryotes including fungi, plants and animals, especially insects and partially mammals. Research concerning their presence in microorganisms, physiological functions, structural characteristics and feasible biotechnological applications has tended to focus on phenol oxidases (POs) of several fungi, especially white-rot fungi [Morozova et al. 2007,Rodriguez-Couta and Toca-Herrera 2006]. In contrast the expression of POs and structurally related non-enzymatic blue multicopper protein structures in prokaryotes has not been so widely investigated [Claus 2003]. As the majority of phenol oxidases described in literature have been isolated from higher fungi, the cellular function for these oxygen-requiring enzymes in eukaryotic systems was typically related to oxidative polymerization and depolymerisation of lignin [Kawai et al. 1988,O'Malley et al. 1993], but also to formation of carposomes linked with synthesis of cell wall-associated pigments [Thurston 1994], sporulation [Leonowicz et al. 2001] and plant pathogenesis [Bar-Nun and Meyer 1989]. Similarities to the occurrence of prokaryotic phenol oxidases can also be considered [Faure et al. 1994] reported prokaryotic PO activity in *Azospirillum lipoferum *which lives, comparable to several soil fungi, in association with the plant rhizosphere and promotes plant growth. This bacterial PO was determined to be expressed in combination with physiological processes like cell pigmentation and the activation of phenolic plant ingredients. Within our previous studies, nitrogen-fixing cultures of the non-symbiotic *Azotobacter chroococcum *SBUG 1484, isolated from composted earth, exhibited PO activity when growing with nutritional deficiencies, especially depletion of exogenous nitrogen sources [Herter et al. 2011]. Interestingly, cell-associated PO production in *A. chroococcum *cells appeared in conjunction with an increased formation of a brown-black pigment identified as melanin. These observations were made concurrently with morphological alteration during the life-cycle of *A. chroococcum *SBUG 1484, in which cell bodies shortened, encapsulated and development of cysts occurred.

Morphologic alterations, formation of dormant stages (particularly spore formation) or production of melanin-like pigments within simultaneous expression of POs or PO-like proteins have also been described for several prokaryotic soil-dwelling bacteria belonging to the genera *Bacillus *[Hullo et al. 2001], *Streptomyces *[Endo et al. 2002], *Pseudomonas *[Mellano and Cooksey 1988], but also *Escherichia *[Kim et al. 2002] and the melanogenic marine bacterium *Marinomonas *[Sanchez-Amat et al. 2001]. Despite common occurrence of these enzymes in similar physiological processes in both prokaryotic and eukaryotic organisms, there still exist remarkable differences with regard to enzymes characteristics and reaction preferences between enzymes not only from both domains, but even POs from species belonging to the same genus. In particular, some prokaryotic POs reveal substrate scopes that overlap with PO-related copper-containing oxidoreductases (tyrosinase) showing polyphenol oxidase (PPO) characteristics, for example the polyphenol oxidase of *Marinomonas mediterranea *[Sanchez-Amat et al. 2001].

Phenol oxidases have been well studied due to their extensive substrate scope and biocatalytic applicability. Studies in bioremediation of xenobiotics [Schultz et al. 2001,Kordon et al. 2010] have shown these enzymes to activate phenolic compounds derived from humic substances or other components with aromatic structures found in soil. Oxidative coupling of non-enzyme substrates with subsequent inactivation and incorporation of formed polymers into soil organic matter [Bollag 1983], presents desirable applications for exploitation of these enzymes diverse functionality.

In this respect the aim of this current study was to provide a preliminary investigation into crude PO activity expressed by the nitrogen-fixing soil bacterium *A. chroococcum *SBUG 1484 through comparative analysis with a fungal PO obtained from the white-rot fungus *Pycnoporus cinnabarinus *SBUG-M 1044 and a tyrosinase (PPO) from the mushroom *Agaricus bisporus*. Therefore, detailed investigation of the substrate scope of the aforementioned enzymes is targeted, to broaden our knowledge of the fundamental characteristics of the cell-associated prokaryotic PO present in crude extract preparations of *A. chroococcum *which are presumably linked to its native cellular function. We aim to understand the effects of enzyme solubilisation and reaction parameters such as influence of metals or inhibitors which may in turn open-up useful applications of this presently wild-type enzyme in the fields of bioremediation and biotechnology, which could then in turn be exploited with future recombinant expression of the enzyme investigated herein.

## Materials and methods

### Cultivation of *A. chroococcum *SBUG 1484

The melanogenic strain *A. chroococcum *SBUG 1484 (Strain Collection, Department of Biology, University of Greifswald) was initially cultivated on solid medium plates of Winogradskis nitrogen-free mineral salt medium as described previously [Herter et al. 2011]. Liquid pre-culture was prepared in 500 mL shake flasks containing 100 mL of sterile Winogradski medium (pH 7.2) supplemented with 1% glucose and 0.4% yeast extract (v/v). Pre-cultures were incubated on a rotary shaker at 30°C and 180 rpm for 12 h. Pre-cultured biomass was harvested by centrifugation (10°C, 20 min, 7,000 × *g*) (Sorvall^® ^RC-53 Refrigerated Superspeed Centrifuge, DuPont Instruments, Bad Homburg, Germany), washed twice with nitrogen-free medium and subsequently transferred into 500 mL flasks containing 100 mL of Winogradskis nitrogen-free medium and 1% glucose (v/v) to reach an initial optical density (OD_500 nm_) of 0.4 for the main cultures.

### Production of bacterial and fungal phenol oxidases (POs)

#### Preparation of crude phenol oxidase from Azotobacter chroococcum cells

After incubation for 56 h (yielding 2.7 ± 0.26 g biomass with a total crude PO activity towards 2,6-DMP of 1.38 ± 0.3 U mL^-1^), cultures were harvested (4°C, 20 min, 7,000 × *g*) and the biomass washed twice with phosphate buffered saline (pH 7.2). The cell pellet was resuspended in 100 mM sodium acetate buffer (NAc buffer) pH 5, and either subsequently disrupted by passing through a pre-cooled French press (SLM AMINCO^®^, Mini-Cell FA-003, Rochester, NY, USA) four times or stored at -20°C until cell lysis. The clarified supernatants obtained after centrifugation (4°C, 2,000 × *g*, 4 min) (Eppendorf AG, Centrifuge 5417R, Hamburg, Germany) of the cell lysates were used as a source of crude phenol oxidase for the herein presented study. Crude extracts were stable without subsequent loss of activity under storage at 4°C. Preparations of outer membrane fraction (containing residues of cell wall debris, cyst walls and Sarkosyl-insoluble outer membrane components) for analytical PAGE were accomplished as described [Herter et al. 2011] after different times of incubation (0, 24, 48, 56 and 72 h).

Solubilisation experiments with detergents, chelating agents and chaotropic reagents were used to anticipate interaction of *A. chroococcum *SBUG 1484 phenol oxidase within the particulate cell debris. For enzyme extraction, non-ionic (Triton X-100, Tween 20, Tween 80), anionic (sodium dodecyl sulfate (SDS), guanidine hydrochloride, sodium lauryl sarcosinate (Sarkosyl), ethylenediaminetetraacetic acid sodium salts (EDTA)) and zwitterionic reagents (3-[(3-cholamidopropyl)dimethylammonio]-1-propanesulfonate (CHAPS)) as well as combinations (Triton X-100/CHAPS, Triton X-100/guanidine hydrochloride, Sarkosyl/guanidine hydrochloride) of each were added to a defined volume of particulate cell debris redissolved in NAc buffer with increasing concentration (5, 10, 20 and 40 mM). When combining detergents, the concentration of CHAPS and guanidine hydrochloride was always set to 5 mM, with increase in molarities of the detergents Triton X-100 and Sarkosyl ranging from 5-40 mM tested. To optimize solubilisation and protein release, lysates were incubated at 30°C for 30 min followed by centrifugation (4,500 × *g*, 2 min) and subsequently, equal volumes of supernatants tested for remaining 2,6-DMP oxidizing activity.

#### Preparation of crude phenol oxidase from Pycnoporus cinnabarinus

The fungus *P. cinnabarinus *SBUG-M 1044 was cultivated for production of the ligninolytic enzyme laccase (PO) as described previously [Jonas et al. 2001,Kordon et al. 2010]. Under the applied culture conditions, *P. cinnabarinus *SBUG-M 1044 produced an extracellular phenol oxidase with an activity of around 0.5 U mL^-1^. After several purification steps including removal of secondary metabolites using DEAE-Sephacel (Sigma, Steinheim, Germany), the eluted enzyme fraction contained crude phenol oxidase which was used for following experiments.

### Enzyme assays

Standard assays were conducted spectrophotometrically (Thermo Fisher UV1 spectrophotometer, Schwerte, Germany) in a total volume of 1 mL containing NAc buffer (100 mM, pH 5) supplemented with 2,6-DMP (5 mM final concentration, λ = 468 nm), and reactions initiated by adding either *A. chroococcum *SBUG 1484 crude phenol oxidase (with a specific activity of 1.18 U mg^-1 ^protein) or *P. cinnabarinus *SBUG-M 1044 crude PO (1.53 U mL^-1^). Examinations with tyrosinase (PPO) were equally performed in sodium phosphate buffer (NaPP, 100 mM, pH 6.5) containing 20 μL of a enzyme stock solution (1 mg lyophilized powder dissolved in 1 mL NaPP, with an activity of ≥1000 U mg^-1^), with monitoring enzyme activity after addition of 5 mM catechol (final concentration, λ = 450 nm) at 25°C. In standard assays, initial rates were determined with individual time-ranges for enzyme preparations (3 min *A. chroococcum *and *P. cinnabarinus *PO, 0.5 min for *A. bisporus *PPO). 1 Unit of activity is defined as the amount of enzyme that catalyzes the conversion of 1 μmol mL^-1 ^min^-1 ^substrate at 25°C.

For determination of optimum pH for catalytic activity of the bacterial crude PO, oxidation of ABTS (5 mM, λ = 420 nm) due to change in absorbance at various pH (pH 1 - 7.5) and in different buffers (maleate, phosphate, acetate, Bis-Tris) was monitored over a time range of 3 min. For examination of thermal stability of *A. chroococcum *SBUG 1484 PO, PO-containing crude extracts were pre-incubated for 30 min in 100 mM NAc buffer (pH 5) at temperatures ranging from 25-55°C, and the remaining activity monitored with ABTS (5 mM) as well as 2,6-DMP (5 mM) at 25°C.

Influence of metal ions and sensitivity to specific organic and inorganic enzyme inhibitors were examined with concentrations from 0.1 to 6 mM. The substrate used for testing of enzyme activity in the presence of repressors and activators was 2,6-DMP (5 mM) for *A. chroococcum *and *P. cinnabarinus *crude PO, whereas tyrosinase (PPO) from *A. bisporus *was monitored using 5 mM catechol.

A comparative analysis of substrate scope for *A. chroococcum *SBUG 1484 PO comprised in crude extracts, *P. cinnabarinus *crude PO and *A. bisporus *PPO was accomplished using seven different groups of model compounds applied for determining laccase and tyrosinase activity. All tested compounds were prepared as 50 mM stock solutions in bidestilled water and examined in 5 mM concentration for enzymatic conversion at wavelengths obtained from literature or deduced from related structures. Substrates were added to initiate the reaction, and the ΔA values of enzymatic oxidation determined spectrophotometrically at various time ranges that were equal for each of the analyzed enzymes with the same substrate and reveal the greatest initial rate of enzymatic oxidation. All experiments were performed with a four-fold repetition to ensure the validity of the results.

### Polyacrylamide gel electrophoresis (PAGE)

For non-denaturing poylacrylamide gel electrophoresis (native PAGE), resolving gels with acrylamide concentrations ranging from 10, 9, 8, 7, 6, 5.5, 5 and 4.5% were used in combination with a 4.5% stacking gel. Samples were mixed in a 2:1 ratio (v/v) with sample buffer (20% glycerol (w/v), 0.0025% bromophenol blue in destilled water) and electrophoresis performed at 20 mA for 2 h in 0.05 M Tris-HCL/0.38 M glycine electrode buffer (pH 8.3). For determination of the relative molecular weight of the herein described bacterial PO from the strain *A. chroococcum *SBUG 1484, native PAGE using a non-denatured protein molecular weight marker kit was conducted. After electrophoresis, proteins were immediately fixed using Coomassie brilliant blue R-25 (5 min), and then shortly washed with distilled water before transferring into 100 mM NAc buffer (pH 5) containing 2,6-DMP for activity staining. Procedures described for characterization of molecular weights in polyacrylamide gels were accomplished according to [Ferguson 1964] and the molecular weight of *A. chroococcum *SBUG 1484 PO was assessed on the basis of its electrophoretic mobility R_f _in comparison to that of the used marker proteins.

Semi-denatured SDS-PAGE was performed as described by [Laemmli 1970] with modifications according to [Solano et al. 2001]. Resolving gels were prepared with a 10% acrylamide concentration and 4% stacking gel. Samples were diluted in the same ratio as already described with sample buffer. After electrophoresis, gels were immediately washed with distilled water and activity staining of bacterial PO was conducted in 100 mM NAc buffer (pH 5) containing different PO substrates such as 2,6-DMP, ABTS, guaiacol and *para*-phenylenediamine. Gels were incubated at 37°C until active proteins became visible. Activity stained protein bands were cut into small slices and added to 100 μL of 100 mM NAc buffer (pH 5). Release of proteins from gel slices was performed by sonication for 2 min. Afterwards, 50 μL of a 2-mercaptoethanol-containing sample buffer was transferred to these samples with heating at 95°C for 15 min. Samples were then analysed by standard SDS-PAGE. Staining of these gels was conducted using the PageSilver™ silver-staining kit (Fermentas, St. Leon-Rot, Germany).

The molecular masses of proteins from semi-denatured and SDS-PAGE were compared using a pre-stained Roti^®^-Mark BICOLOR marker (Roth, Karlsruhe, Germany) and a low molecular weight range marker (Sigma, Steinheim, Germany).

### Chemicals

All chemicals were purchased from Sigma-Aldrich (Taufkirchen, Germany). Vanillin azine and *ortho*-vanillin were obtained from Acros Organics (Geel, Belgium); dopamine hydrochloride was purchased from Alfa Aesar^® ^(Karlsruhe, Germany). Eugenol was acquired from ABCR (Karlsruhe, Germany) and 3-isopropylcatechol from ChemService, Inc. (West Chester, Pennsylvania, USA). Lyophilized tyrosinase (EC 1.14.18.1) from mushroom (*A. bisporus*) and a non-denatured protein molecular weight marker kit were purchased from Sigma-Aldrich (Steinheim, Germany).

## Results

### Release of phenol oxidase from particulate cell fraction

Phenol oxidase (PO) activity had previously been determined in nitrogen-fixing cells of the strain *A. chroococcum *SBUG 1484 which undergo encystment and a melanogenic change in appearance [Herter et al. 2011]. Examinations concerning the localization of PO in culture supernatants and various cellular fractions revealed strong association within particulate cell debris, especially outer membrane fractions containing residues of cell and cyst walls [Herter et al. 2011] and therefore comparably minor amounts were observed to be released into supernatants of enzyme preparations. With respect to the herein performed experiments using crude phenol oxidase initial attempts to purify the native enzyme contained within *A. chroococcum *crude extracts were not successful due to irreversible absorption of this enzyme to a wide range of columns and membranes under a variety of conditions. Therefore, instead of using mandatory purified enzyme preparation for comprehensive enzyme characterization studies, experiments were conducted with crude phenol oxidase aiming to provide an initial understanding of general characteristics of this particular PO.

With regard to this, solubilisation experiments with different detergents were conducted in this current study to monitor the release of cell-associated bacterial phenol oxidase from particulate cell debris for determining effects on activity. For enzyme extraction, non-ionic, anionic and zwitterionic reagents as well as combinations of each were added at various concentrations (5 mM up to 40 mM). With reference to the nature and target location of the detergents used for solubilisation of proteins, integration of laccase in particulate cell fractions could be estimated and elevated release into crude extracts realized.

**Figure 1:**
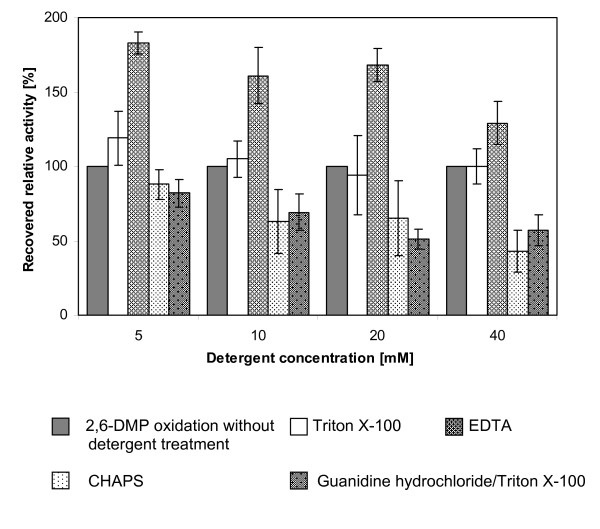
**Treatment of particulate cell debris with detergents, chelating agents and chaotropic reagents**. Recovered activities of supernatants before (100% relative activity) and after incubation of particulate cell debris (obtained from a 56 h-cultivation) with selected solubilising detergents in increasing concentrations for 30 minutes, monitored by oxidation of 2,6-DMP. Error bars refer to standard deviation by means of four replicates.

The greatest amount of recovered PO activity in supernatants (6,000 × *g*) was monitored by using the anionic detergent EDTA (5 mM) (Figure 1). A 20-30% recovery in 2,6-DMP oxidizing activity within supernatants was also determined with the addition of 5-10 mM Triton X-100. Other detergents and mixtures of non-ionic, anionic or zwitter-ionic solubilising, chaotrophic and chelating agents showed negligible positive effects, whilst some were also found to decrease the observed crude PO activity (Figure 1). In the presence of linear chained sodium dodecyl sulfate (SDS) no activity was present after treatment even at the lowest concentration, indicating that denaturation of protein and loss of its native state had occurred. The same results were found using the long alkyl side-chained Tween 20, whereby 5 mM of detergent addition reduced activity to 33%. The addition of Tween 80 at the same concentration also resulted in no residual oxidation activity.

### Effect of pH and temperature

The effect of pH on crude phenol oxidase activity was monitored with the canonical substrate ABTS in different buffers ranging in 0.5 steps from pH 1 to 7.5, with the highest activity observed around pH 4.5-5.0 in NAc buffer (100 mM) (see Additional file 1). Based upon the pH optimum, thermal stability was also tested using NAc buffer at pH 5 in a temperature range from 25-55°C (see Additional file 2). In the case of ABTS oxidation, crude PO showed highest activity when pre-incubated at 35°C, with 118% activity relative to that recovered with 2,6-DMP at the same temperature. Activity at temperatures above 50°C (60% residual activity) could not be monitored further due to substrate autoxidation. Oxidation of 2,6-DMP was optimal with pre-incubation of crude phenol oxidase preparations between 25°C and 30°C but simultaneously decreased with increasing temperatures, whereby 70% of activity was recovered after incubation at 50°C. Nevertheless with regard to pre-incubation periods of 30 min, the examined phenol oxidase derived from crude cell extracts showed remarkable thermal stability.

### Influence of metals

The effects of metal ions (concentrations 0.01, 0.1, 1 and 2 mM) on 2,6-DMP oxidizing capacity of the herein examined bacterial crude phenol oxidase are summarised in Table 1. Examinations into the influence of CuSO_4_, CuCl_2_, FeCl_3_, NiCl_3 _and (CH_3_COO)_2_Pb were conducted with the substrate ABTS as they were observed to stimulate strong autoxidation of 2,6-DMP. *A. chroococcum *SBUG 1484 crude PO showed strongest sensitivity towards iron sulfate, where at a concentration of 0.1 mM, a 75% reduction in activity was observed. In contrast, trivalent iron ions (FeCl_3_) did not show the same inhibiting effects at the tested concentrations. Addition of 1 mM ZnSO_4_, H_3_BO_3 _and (CH_3_COO)_2_Pb revealed crude PO inhibition with 60% remaining activity, whereas the presence of MnSO_4_, CoCl_2 _and CaCl_2 _showed lower inhibitory effects with relative activities around 80%. In the presence of 0.1 mM Mg^2+ ^and Cu^2+ ^salts, positive effects on oxidizing activity could be monitored with increases in recovered activity of up to 10%. Remarkable enhancement of activity was achieved with the addition of 1 mM CuSO_4 _with an increased relative activity of 130%. Slight stimulation of crude PO activity was also determined with the addition of NiCl_3 _and NaMoO_4_.

**Table 1:** Comparative study of activating or inhibiting influences caused by metal ions, on the crude phenol oxidase activity of *A. chroococcum *SBUG 1484 towards the substrate 2,6-DMP (5 mM)

Metal compound^a^	Concentration (mM)	Relative activity [%]
None		100

ZnSO_4_	0.1	84
	1.0	61
	
FeSO_4_	0.01	84
	0.1	25
	
MnSO_4_	0.1	89
	1.0	87
	
MgSO_4_	0.1	110
	1.0	99
	
CuSO_4 _	0.1	108
	1.0	130
	
MnCl_2_	0.1	104
	1.0	86
	
CoCl_2_	0.1	98
	1.0	80
	
CaCl_2_	0.1	90
	1.0	85
	
CuCl_2_	0.1	112
	1.0	78
	
FeCl_3 _	0.1	108
	1.0	79
	
NiCl_3_	0.1	109
	1.0	106
	
NaMoO_4_	0.1	105
	1.0	106
	
H_3_BO_3_	0.1	85
	1.0	68
	
(CH_3_COO)_2_Pb	0.1	77
	1.0	64

Shown results are the average of four replicates.

^a ^Effects of CuSO_4_, CuCl_2_, FeCl_3_, NiCl_3 _and (CH_3_COO)_2_Pb were in contrast to other tested metals determined by the oxidation of 5 mM ABTS, further conditions were kept constant.

### Effect of organic solvents on crude phenol oxidase activity

**Figure 2:**
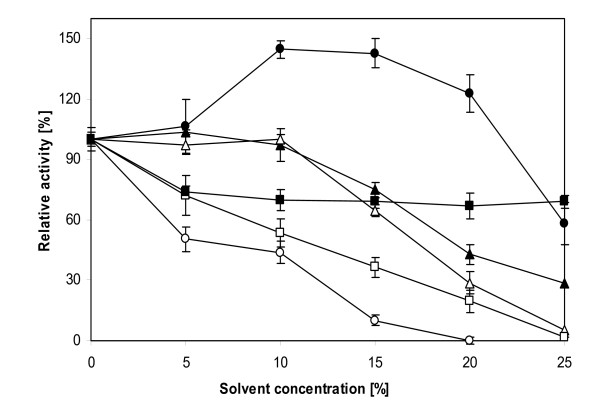
**Effect of organic solvents on crude phenol oxidase activity**. 2,6-DMP oxidizing activity after incubation of *A. chroococcum *SBUG 1484 crude PO preparations (crude extracts obtained from a 48 h-cultivation) for 60 minutes at 30°C in the presence of *n*-hexane (filled square), THF (open square), DMSO (closed circle), acetonitrile (open circle), ethanol (closed triangle) and methanol (open triangle) at different concentrations. Activities were calculated relative to 2,6-DMP oxidation in the absence of organic solvents.

2,6-DMP oxidizing activity was determined after PO-containing crude extracts were incubated (20 min and 60 min at 30°C) with aprotic unpolar solvents (*n*-hexane, tetrahydrofuran (THF)), aprotic polar solvents (dimethylsulfoxide (DMSO), acteonitrile) and the protic solvents ethanol and methanol. All solvents were tested in 5 percent steps from 5% up to 35% solvent concentrations. Generally, when incubating crude enzyme preparations in presence of solvents, the inhibitory effects determined after 20 min were found to be compensated over incubation time of 60 min. Incubation of crude phenol oxidase in the presence of 15% solvent concentration revealed 69% (*n*-hexane) and 38% (THF) relative activities after 60 min (Figure 2). When acetonitrile was applied as solvent, a balanced time-dependent effect could not be monitored with remaining activities of 23% (20 min) and 10% (60 min). In contrast to this, DMSO which belongs to the same solvent classification, provokes enhancement in relative activities, whereby at a concentration of 15%, 2,6-DMP oxidizing activity remains relatively high at 110% (20 min) and 142% (60 min) respectively. With a view to solvent classification and the herein tested examples, protic solvents such as ethanol and methanol revealed the lowest inhibitory effects on crude PO activity with relative activities of 77% (ethanol) and 63% (methanol) after 60 min incubation at solvent concentration of 15% (Figure 2).

### Comparative assessment within inhibitor studies

**Figure 3:**
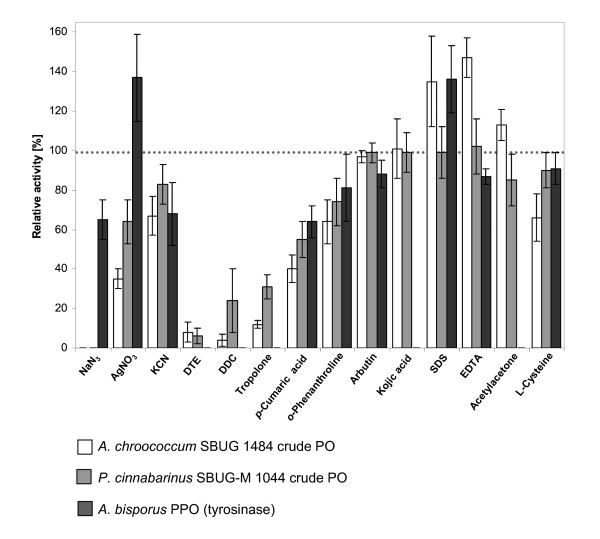
**Inhibitory studies on PO and PPO activities**. Comparison of the effects of general enzyme inactivators and selected PO or PPO inhibitors on *A. chroococcum *SBUG 1484 crude PO, *P. cinnabarinus *SBUG-M 1044 crude PO and *A. bisporus *PPO (tyrosinase) in 2 mM concentrations. Effects on bacterial and fungal PO activities were evaluated using 5 mM 2,6-DMP, whereas PPO activity in the presence of potential inhibitors was monitored in the oxidation of catechol (5 mM). Error bars refer to standard deviation by means of four replicates.

Sodium azide (0.1 mM, NaN_3_) was identified as the most inhibiting substance of all tested inhibitors towards *A. chroococcum *SBUG 1484 as well as *P. cinnabarinus *SBUG-M 1044 crude PO activity, whereas tyrosinase of *A. bisporus *still showed 65% relative activity in the presence of 2 mM NaN_3 _(Figure 3). Diminishment in catechol oxidizing activity of *A. bisporus *PPO in the presence of NaN_3 _was observed along with a slight reddish colouration of assay mixtures. This change in colouration differed to the common yellowish reaction mixtures and was clearly evoked by addition of PPO and not produced by non-enzymatic reaction between both substances. Further remarkable decrease in activity up to 35% of *A. chroococcum *PO comprised in crude extracts was monitored with the inorganic enzyme inhibitor silver nitrate (AgNO_3_), whereby crude PO of *P. cinnabarinus *revealed a two-fold higher remaining activity. Addition of organic inhibitors such as the disulfide-bond cleaving agent dithiothreitol (DTE) and copper chelator diethyldithiocarbamic acid (DDC) revealed strong decreases in 2,6-DMP oxidizing activities of both PO preparations and gave complete inactivation of fungal PPO. The metal chelator tropolone also gave a strong negative influence on bacterial and fungal PO activity (12% and 31% remaining activity respectively), and was found to completely inhibit *A. bisporus *PPO (Figure 3). Inhibitory effects of the metal chelator *p*-coumaric acid were less pronounced on the tested crude POs and PPO with remaining relative activities ranging from 40% to 64%. In contrast, the presence of 2 mM kojic acid and arbutin (tyrosinase inhibitors) gave only negligible inhibitory effects on *A. chroococcum *PO (3% inhibition) as well as fungal PO (1% inhibition), whereas catechol oxidizing activity of *A. bisporus *PPO was reduced to 50% relative activity. The addition of SDS showed remarkable enhancement of 2,6-DMP oxidizing activity of *A. chroococcum *crude extracts and *A. bisporus *PPO up to 135%, whereas activity of *P. cinnabarinus *PO preparation was relative unaffected by the addition of 2 mM SDS (Figure 3). EDTA could be monitored as a suitable activator of bacterial and fungal crude PO activity, whereas PPO activity was increasingly inhibited with greater concentrations of this organic acid. The addition of acetylacetone increased activity of *A. chroococcum *crude PO, however it was monitored to slightly inhibit activity of *P. cinnabarinus *PO preparations and completely inhibit *A. bisporus *PPO (Figure 3). In the presence of 2 mM L-cysteine, the oxidizing activity of *A. chroococcum *PO preparations was noticeably reduced, whereas crude PO of *P. cinnabarinus *and PPO of *A. bisporus *showed only a marginal 10% decrease in activity. Generally, the herein examined bacterial crude PO of the strain *A. chroococcum *SBUG 1484 reacted in the presence of selected inhibiting inorganic and organic agents in a manner comparable to that of fungal PO preparations from *P. cinnabarinus *SBUG-M 1044. Consequently clear differences to PPO activity using tyrosinase from *A. bisporus *were observed (Figure 3).

### Comparison of substrate acceptance

As the crude PO derived from *A. chroococcum *SBUG 1484 cell-free extracts could be found to express real laccase activity based upon its substrate spectrum and without necessity of further activation by copper-supplementation [Herter et al. 2011], experiments were conducted to obtain an extended depiction compared to the substrate spectrum of an fungal crude PO preparation exhibiting the same pH optimum (*P. cinnabarinus*, pH 5.0) and tyrosinase (PPO) of mushroom (*A. bisporus*, pH 6.5). Within this, seven different groups of potential substrates were tested with group 1-substances structurally derived from guaiacol, including mono-methoxylated (and methylated) monophenols. Group 2 (dimethoxylated monophenols) consisted of substrates related to 2,6-DMP, which was examined as the most preferred aromatic substrate of *A. chroococcum *crude PO preparations. Groups 3 and 4 included *ortho*- and *para*-dihydroxylated compounds that were defined as typical catecholase substrates (3) and laccase substrates (4) respectively. In addition to melanin-precursors (group 5), *meta*-dihydroxylated alkylresorcinols (group 6) as well as exclusive PO and PPO substrates (group 7) were examined in this analysis. Comparison of the results obtained with crude bacterial and fungal phenol oxidase as well as fungal polyphenol oxidase are summarised in Table 2.

**Table 2:** Examination of substrate preferences towards model compounds exhibited by PO containing cell-free extract from *A. chroococcum *SBUG 1484 compared to fungal crude PO preparations from *P. cinnabarinus *SBUG-M 1044 and PPO from *A. bisporus*

	Initial rate of activity (ΔA min^-1 ^mL^-1 ^× factor 1,000)			
**Model compound**	**Wavelength** **[nm]**	***A. chroococcum *PO**	***P. cinnabarinus *PO**	***A. bisporus *PPO**

**Compound group 1**				

Guaiacol	436	10	28	2
*para*-Vanillin	436	0^a^	0	0
*ortho*-Vanillin	468	40	28	0
Vanillin acid	436	0	0	0
Vanillin alcohol	420	42	0	0
Vanillin azine	468	0	6	0
4-Hydroxy-3-methoxy-*α*- methylbenzyl alcohol	420	24	0	0
2-Methoxy-6-methylphenol	468	54	0	0
2-Methoxy-4-methylphenol	420	34	0	0
2-Methoxy-4-propylphenol	420	84	0	0
2-Hydroxy-6-methoxybenzaldehyde	420	0	0	0
2-Hydroxy-5-methoxybenzaldehyde	420	36	4	0
Eugenol	420	76	32	0
Ferulic acid	480	0	0	0

**Compound group 2**				

2,6-Dimethoxyphenol (2,6-DMP)	468	746	532	20
2,3-Dimethoxyphenol	468	5	0.6	0.2
Syringic acid	468	0.8	0.4	0.4
Syringaldehyde	468	1	0.4	0.2
4-Methyl-2,6-dimethoxyphenol	468	0.6	0.2	0
2,6-Dimethylphenol	468	0.7	1.8	0.6
Syringaldazine	525	0	22.4	0

**Compound group 3**				

Catechol	450	9	2.5	934
3-Methylcatechol	420	29	30	412
4-Methylcatechol	420	24	8	572
*tert*-Butylcatechol	420	13	8	886
3-Methoxycatechol	420	13	9	52
3-Isopropylcatechol	420	34	20	362
Hydrocaffeic acid	530	1.5	0	74

**Compound group 4**				

Hydroquinone	285	0.5	1	0
2-Methylhydroquinone	285	0.5	1.2	0.2
2-Methoxyhydroquinone	285	0.8	2.4	1.2
*tert*-Butylhydroquinone	285	0.4	1.6	0.8
2,3-Dimethylhydroquinone	400	0.8	0.6	0
2,6-Dimethoxyhydroquinone	400	2.6	3	0

**Compound group 5**				

4-Hydroxyindole	400	5	6	4
3-(3,4-Dihydroxyphenyl)-L-alanine	475	4	3	388
Tyrosine^b^	280	0	0	6
Dopamine	400	8	2.3	448
3,4-Dihydroxybenzoic acid	400	0.6	2	8
3,4-Dihydroxyphenylacetic acid	400	1	3	1,072

**Compound group 7**				

ABTS	420	582	638	18
Pyrogallole	450	108	14	170
*para*-Phenylenediamine	523	78	90	30
*para*-Cresol	300	0	0	8

Initial rate of enzyme activity was measured with values shown multiplied by a factor of 1,000.

^a ^Change in absorbance could not be determined within prolonged incubation times, highlighting the compound as a non-enzyme substrate under the conditions applied.

^b ^Assaying the oxidation of tyrosine (compound group 5) and *p*-cresol (compound group 7) was accomplished after a pre-incubation period of 5 minutes at 25°C.

With group 1 compounds, preference of *A. chroococcum *crude PO towards methoxy-substituted monophenols was confirmed. Generally, model compounds that reveal a methoxy-substituent in position 2 could be described as *A. chroococcum *crude PO preferred substrates. Activity in crude extract preparations was enhanced when at position 6 of the aromatic ring a further methyl substituent was present (e.g. 2-methoxy-6-methyl phenol). With tyrosinase, none of the tested substrates except guaiacol were found to be oxidized. However, *P. cinnabarinus *PO crude preparation showed significant activity towards guaiacol and was also found to oxidize *ortho*-vanillin, 2-hydroxy-5-methoxy benzaldehyde and eugenol. This fungal crude PO was also active in vanillin azine oxidation, whereby PO-containing crude extracts of *A. chroococcum *failed in the oxidation of this particular substrate.

With 2,6-DMP belonging to group 2 compounds (dimethoxylated monophenols) bacterial crude PO revealed the highest activity towards all tested substrates of this class followed by oxidation of 2,3-dimethoxyphenol. All tested substances were also found to belong to the *P. cinnabarinus *crude PO substrate spectrum as they were marginally converted by fungal tyrosinase with exception of 4-methyl-2,6-dimethoxyphenol. As with the group 1-compound vanillin azine, syringaldazine which is structurally deduced from 2,6-DMP was only oxidized by *P. cinnabarinus *PO preparations.

*Ortho*-dihydroxylated substrates (group 3) were converted with highest rates using tyrosinase from mushroom, with activities typically between 2-8 fold higher than those of bacterial and fungal crude PO preparations. This confirms the distinctive feature of PPOs, especially tyrosinase, in exhibition of catecholase activity, and enhances the link to POs that were described for their ability in oxidizing *ortho*-dihydroxylated compounds. When comparing *A. chroococcum *PO-comprising crude extracts and *P. cinnabarinus *PO preparations, similar activities towards methyl-, methoxy- and alkyl substituted catechols were determined. Hydroquinoid substrates belonging to group 4 that were designated as substrates for explicit differentiation of PO activity from PPO activity were found to be oxidized by *A. chroococcum *as well as *P. cinnabarinus *crude PO. In this respect, tyrosinase was interestingly determined to be active in the oxidation of 1,4-dihydroxylated compounds such as methylhydroquinone, methoxyhydroquinone and *tert*-butylhydroquinone. Despite the aforementioned differences of POs and PPOs on the basis of activity towards 1,2- and 1,4-dihydroxylated substrates, with the examined model compounds of group 4 in conjunction with bacterial and fungal crude PO; we observed an extended time range in which measurements must be conducted due to lower activities when compared to *ortho*-dihydroxylated substrates. Generally, 2,6-dimethoxy-hydroquinone was the most preferred compound of *A. chroococcum *crude PO as well as *P. cinnabarinus *PO preparations, whereby other tested hydroquinoid compounds revealed two to seven-fold diminished activity for PO-containing extracts of *A. chroococcum*. With melanin-precursors and related compounds (group 5) *A. chroococcum *crude PO was able to activate the monophenolic compound 4-hydroxyindole as well as 3-(3,4-dihydroxyphenyl)-L-alanine (L-DOPA) and dopamine with comparable activity, whereby fungal PO preparations revealed slightly lower values. In comparison to both POs, tyrosinase from mushroom exhibits up to seven times increased activity rates when using L-DOPA and dopamine as substrates. This observation confirms that these *ortho*-dihydroxylated compounds are appropriate substances for determining catecholase activity expressed by tyrosinases, whereby tyrosine is the sole substrate that was converted by mushroom tyrosinase during the herein described spectrophotometric examinations. As expected, activity towards tyrosine could not be monitored with *A. chroococcum *and *P. cinnabarinus *enzyme preparations.

Resorcinol and related *meta*-dihydroxylated alkylresorcinols (group 6) were not found to be oxidized by any of the tested members belonging to the enzymatic classes of POs and PPOs.

Referring to group 7 compounds, extracts containing *A. chroococcum *PO were examined to show highest preference towards the non-phenolic substrate ABTS, with activity values comparable to those gained with 2,6-DMP. With *P. cinnabarinus *PO, slightly higher oxidation rates could be determined whereby this substrate gave the greatest activity values of all tested compound groups. With ABTS oxidation, tyrosinase from mushroom showed in comparison to the researched POs a three-fold reduced rate of conversion; however using pyrogallole it was found to express a two-fold greater oxidation rate than *A. chroococcum *crude PO. The aminated non-phenolic compound *para*-phenylenediamine was oxidized equally by *A. chroococcum *and *P. cinnabarinus *PO preparations. Activity towards *para*-cresol was only observable in assay mixtures with tyrosinase, confirming that PO of *A. chroococcum *exhibits no PPO characteristics by means of a cresolase activity.

Based upon the screening of potential PO substrates in comparison to known PPO substrates, the following range of appropriate substrates for *A. chroococcum *PO present in crude extracts could be constructed based upon activity observations: 2,6-DMP ≥ ABTS >*ortho*-dihydroxylated compounds ≥ mono methoxylated monophenols > dimethoxylated monophenols >*para*-dihydroxylated compounds. Lowest levels of activity with crude PO preparations from *P. cinnabarinus *SBUG-M 1044 were also observed when using *para*-dihydroxylated compounds as substrates.

### Analytical PAGE

**Figure 4:**
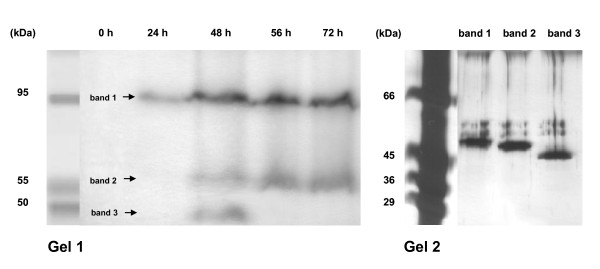
**Semi-denaturing and SDS-PAGE analysis of outer membrane preparations obtained from *A. chroococcum *SBUG 1484 cells incubated under nitrogen-fixing conditions**. Gel 1: Semi-denaturing PAGE of outer membrane preparations (0 - 72 h cultivation) containing residues of cell wall debris, cyst walls and sarkosyl-insoluble outer membrane components stained for PO activity with *p*-phenylenediamine at 37°C for 10 minutes in NAc buffer (100 mM, pH 5). Arrows reference *p*-phenylenediamine oxidizing protein bands 1-3. Molecular weights were estimated using a pre-stained Roti^®^-Mark BICOLOR marker. Gel 2: Silver-staining of excised and completely denatured *p*-phenylenediamine active proteins (48 h sample) subsequently analyzed via SDS-PAGE. Examination of molecular weights of extracted proteins was accomplished using a low range molecular weight marker (14 - 66 kDa).

Analysis of the relative molecular weight of *Azotobacter *PO was accomplished indirectly in a non-denaturing system according to [Ferguson 1964] with crude extract preparations. The migration distances of the 2,6-DMP stained protein band in coherence with those of marker proteins were recorded on gels comprising eight different acrylamide concentrations. Calculating the electrophoretic mobility (R_f_) facilitated creation of linear plots, whereby the molecular weight of the PO was calculated as 142 kDa (data not shown). This indicates that *A. chroococcum *PO acts as a homotrimer under non-denaturing conditions as already postulated previously [Herter et al. 2011]. Activity staining of the identical preparations run under semi-denaturing conditions revealed one 2,6-DMP active band at a apparent molecular weight between 40 and 45 kDa (data not shown). When conducting semi-denaturing PAGE with samples obtained from outer membrane preparations (comprised of cell and cyst walls) after different incubation times (0 h, 24 h, 48 h, 56 h and 72 h) and staining for activity with specific PO indicators (2,6-DMP, ABTS, guaiacol and *para*-phenylenediamine), results were revealed to be much more complex than in turbid supernatant samples gained from crude extracts (Figure 4, Gel 1). With all samples one defined redox-indicator active band occurred at an estimated molecular weight of 95 kDa (band 1), whereby in case of the 24 h-sample it was the only band identified at this incubation time. With the exception of the 24 h-sample, all other time-points gave a further active band at approximately 55 kDa (band 2). Interestingly within the 48 h-sample an additional active band at 50 kDa was also determined (band 3). Active bands 1-3 were excised and the protein extracted before subjecting to SDS-PAGE analysis followed by silver-staining. This showed all bands to posses approximately the same molecular weight around 45 kDa with only minor differences of 5 kDa (Figure 4, Gel 2). These differences may be due to factors such as varying extents of glycosylation [Perry et al. 1993], and could be linked to the conditions of sample preparation or the localization of PO in different fractions of outer membrane preparations (containing residues of cell and cyst wall) or proteolytic modification. However, performing normal SDS-PAGE analysis revealed within all samples, one Coomassie stained band with an apparent molecular weight in the range of 45 kDa (data not shown).

## Discussion

In the present study, an incipient approach on general properties exhibited by the cell-associated bacterial PO comprised within crude PO preparations of nitrogen-fixing cells from the strain *A. chroococcum *SBUG 1484 was performed. As prior attempts in preparation of purified enzyme were not successful, the experiments performed herein focussed on an initial understanding of basic enzymatic properties of this prokaryotic crude phenol oxidase, such as substrate scope and the influence of inhibitors, metals and solvents.

Solubilisation experiments with particulate cell debris were only effective with anionic EDTA and the non-ionic agent Triton X-100. EDTA was identified as being the most effectual agent in protein release, indicating that *A. chroococcum *SBUG 1484 crude phenol oxidase may act as a peripheral protein with electrostatic or ionic interactions to membrane lipids (occurring in *A. chroococcum *within the cytoplasmic membrane, outer membrane, and compounds of cyst walls) mediated by metal ions, particularly calcium. Through addition of EDTA to cyst or membrane fractions, lipopolysaccharides which are usually stabilised by divalent cations such as Ca^2+^, Mn^2+ ^and Mg^2+ ^become more fluid, resulting in permeabilization and subsequent release of proteins due to the formation of EDTA-metal complexes [Goldschmidt and Wyss 1966]. Furthermore, the addition of EDTA reduces the enzymatic inhibition typically caused by heavy metal ion stimulated oxidation of thiol-groups by molecular oxygen. A slight increase in enzyme activity and protein release could also be monitored when preparing membranes with Triton X-100. This detergent shows high affinity to hydrophobic regions, especially phospholipid bilayer membranes whereby effective binding and solubilisation of phospholipids from cytoplasmic membrane portions of cell wall fragments are commonly described [Reisinger and Eichacker 2006]. Herein we found *A. chroococcum *crude PO to exist as a native active homotrimer (142 kDa), which is also active in monomeric and dimeric states. Accordingly, PO of *Streptomyces psammoticus *was found to act as a monomer with an apparent molecular weight of 45 kDa [Niladevi et al. 2008], whereby *Streptomyces griseus *PO showed comparable results to *A. chroococcum *SBUG 1484 with an active native homotrimer comprised of 38 kDa monomers [Endo et al. 2003]. Smaller prokaryotic phenol oxidases between 30 and 70 kDa are generally observed, whilst fungal laccases tend to exhibit higher molecular weights ranging from 70 kDa for *P. cinnabarinus*, *Aspergillus niger *and *Aspergillus oryzae *PO [Sigoillot et al. 2003] up to 100 kDa for monomeric structures. PO of *A. chroococcum *SBUG 1484 in cell-free extracts revealed a pH optimum of 4.5 when oxidizing the substrate ABTS. Together with a beneficial pre-incubation at 35°C and 25-30°C (2,6-DMP, pH 5.0) respectively, parallels can be drawn to phenol oxidases and laccase-like enzymes especially expressed by other soil bacteria known for establishment of dormant stages during their life-cycle [Durao et al. 2008]. Crude PO from strain SBUG 1484 was determined as not requiring exogenous copper sources for activation, as it has been described previously for pseudo laccases [Solano et al. 2001]. Copper has often been described in stimulating the oxidizing activity of prokaryotic phenol oxidases, for example CueO of *E. coli *[Li et al. 2007]. This can be related to the metal-coordinating characteristics of laccase, and other related copper-containing oxidases which are structurally different to fungal laccases. However, copper activation of laccases is not constricted on bacterial POs, as this metal was also determined to activate fungal PO, for instance *Daedalea quercina *laccase [Baldrian 2004].

The application of enzyme-mediated synthesis of fine chemicals is accepted as an effective and preferred alternative towards chemical synthetic routes. Non-aqueous enzymology by means of medium-engineering has been proposed as alternative to common biotransformation reactions in industry [Stöcklein and Scheller 1995], and with a view to increasing applicability, the herein described crude PO from *A. chroococcum *SBUG 1484 was tested for activity in various organic solvents often used in biotransformation and biocatalysis. Generally, DMSO was determined as the most activity enhancing solvent presumably allowing the PO to retain its active confirmation [Tominaga et al. 2004], whereas the protic solvents ethanol and methanol gave minimal inhibitory effects on activity. However, greatest decrease of *Azotobacter *PO activity recovered in crude extracts was monitored through addition of 15% acetonitrile, even though it is an aprotic polar solvent like DMSO. The noticeable degree of solvent stability, which is displayed by *A. chroococcum *crude PO, raises potential future application of this enzyme in bioremedial and biotechnological processes of which organic solvents can often be found as a co-solvent.

In order to estimate the native ecological role of this prokaryotic enzyme, primary biochemical investigations were conducted in an attempt to explore substrate scope in comparison to fungal crude PO preparations of *P. cinnabarinus *SBUG-M 1044 and PPO (tyrosinase) of *A. bisporus*. A clear preference of *A. chroococcum *SBUG 1484 PO-containing crude extracts was determined towards *ortho*-dihydroxylated compounds followed by mono-methoxylated monophenols, and dimethoxylated compounds (except 2,6-DMP with the highest activity of all substrates tested). Diminished activity towards *para*-dihydroxylated compounds was somewhat surprising as POs are strictly differentiated from catecholases and tyrosinases due to an exclusive and pronounced oxidation of 1,4-dihydroxylated compounds [Xu 1996]. However crude PO preparations from *P. cinnabarinus *SBUG-M 1044 showed comparable results to those obtained for *A. chroocoocum *crude phenol oxidase preparations. According to [Solano et al. 2001], results in the case of *para*-dihydroxylated substances could be reasoned with low molar adsorption coefficients at visible regions and instability of the formed products, suggesting *para*-diphenols as non-appropriate substrates in spectrophotometric standard assays. Substrates belonging to seven different compound classes were equally tested in a 5 mM concentration (2 mM for syringaldazine), which in some instances may lead to substrate inhibition of the tested enzymes. The pronounced oxidative activity of *A. chroococcum *SBUG 1484 crude PO towards 2,6-DMP and ABTS, a feature shared with *P. cinnabarinus *SBUG-M 1044 preparations, clearly confirms laccase-type characteristics of the herein described *Azotobacter *phenol oxidase. Furthermore, the studied bacterial PO comprised in cell-free crude extracts exhibited no PPO activity towards oxidation of tyrosine and *para*-cresol, which was however clearly determined with *A. bisporus *PPO. Crude PO of *A. chroococcum *SBUG 1484 possesses 128% relative activity towards 2,6-DMP when compared to ABTS [Niladevi et al. (2008)]. studied 2,6-DMP as the preferred substrate for *S. psammoticus *PO with 214% activity relative to that with ABTS. Despite the similarity between *A. chroococcum *SBUG 1484 and *P. cinnabarinus *SBUG-M 1044 crude PO preparations in 2,6-DMP oxidation, the examined prokaryotic PO differentiates from fungal PO due to an inability to oxidize vanillin azine and the typical laccase substrate syringaldazine. Both of these substrates reveal methoxy-substituents with an azine bridge at the C_4_-carbon atom of the aromatic ring. Structurally similar compounds, with methyl-, methylen or hydroxy-groups at the C_4_-position did not reduce activity of *A. chroococcum *crude PO, whereas substrates containing an acid or aldehyde residue as the terminal group were not oxidized. *A. chroococcum *SBUG 1484 as well as *P. cinnabarinus *SBUG-M 1044 PO preparations revealed no activity towards *meta*-dihydroxylated compounds, which has also been observed in many other POs with resorcinol derived substrates [Palmieri et al. 1997]. In case of *ortho*-diphenols the herein examined crude POs can be distinctly distinguished from PPO activity on the basis of decreased ΔA values within an extended time-range in which highest initial rates were obtained. However, comparing activity of *A. chroococcum *SBUG 1484 crude PO towards catechol-derivatives with other tested compounds, a high affinity towards substituted catechols was estimated. Laccase activity towards both, *ortho*- and *para*-dihydroxylated compounds has also been previously reported [Xu 1996].

Through examining activity of *A. chroococcum *SBUG 1484 crude PO we have been able to describe remarkable differences in substrate specificity of prokaryotic POs, and also between prokaryotic and eukaryotic POs; and which may also enable reconsideration of previously designated bacterial and fungal PO activities. Studies on the substrate spectrum of *A. chroococcums *crude PO indicate that this enzyme could be useful in biotransformations involving mono- and dimethoxy-substituted phenols as well as *ortho*-diphenolic compounds.

As *Azotobacter *is a well-studied soil bacterium interacting with plant rhizosphere and evoking improved plant growth due to cells nitrogen-fixing activity and production of plant growth regulators, there may exist possible physiological advantages when expressing PO activity, which is closely related in terms of the determined substrate preferences related to (methoxylated) plant phenolic compounds. As enzyme preparations of *A. chroococcum *revealed activity towards selected melanin-precursors, PO expression can be linked to synthesis of melanin and the concurrent consumption of oxygen during pigmentation of this prokaryote. Therefore, besides its assumed relevance in interaction with plants and compounds which can be derived there from; oxygen-requiring PO of *A. chroococcum *SBUG 1484 may play a contributory role in protection of oxygen-sensitive nitrogenase, besides a high inner respiratory rate, and which evokes an enhanced rate of oxygen consumption. A detailed kinetic analysis of this enzyme will be facilitated by future investigation into cloning and recombinant expression. Together with the knowledge gained herein as to the preferred substrates of this PO and the effects of various solvents and reagents, the application of this enzyme as a potential valuable biocatalyst is one step nearer.

## Competing interests

The authors declare that they have no competing interests.

## Supplementary Material

Additional file 1 **pH optimum of *A. chroococcum *crude PO preparation towards oxidation of ABTS**. Relative activities of *A. chroococcum *crude PO preparation in different buffers ranging from pH 1 to 7.5 monitored due to the oxidation of 1 mM ABTS. Substrate oxidation in pH ranging from 1 to 3 was examined using maleate buffer, whereas activities from pH 3.5 to 6 were monitored in sodium acetate buffer (100 mM) and pH 6 to 7.5 in 100 mM phosphate-citrate buffer. Click here for file

Additional file 2 **Thermal stability of *A. chroococcum *PO comprised in crude extracts monitored with ABTS and 2,6-DMP**. Thermal stability determined with ABTS (filled square) and 2,6-DMP (filled triangle), with samples pre-incubated for 30 minutes in NAc buffer (100 mM, pH 5) at temperatures ranging from 25-50°C. Error bars refer to standard deviation by means of four replicates. Click here for file
