# Supplementary material for: Study of enzymatic properties of phenol oxidase from nitrogen-fixing Azotobacter chroococcum
Source: AMB Express. 2011 Jun 24;1:14. doi: 10.1186/2191-0855-1-14 (PMC3402154; doi:10.1186/2191-0855-1-14)
Supplement: Additional file 1 — pH optimum of A. chroococcum crude PO preparation towards oxidation of ABTS. Relative activities of A. chroococcum crude PO preparation in different buffers ranging from pH 1 to 7.5 monitored due to the oxidation of 1 mM ABTS. Substrate oxidation in pH ranging from 1 to 3 was examined using maleate buffer, whereas activities from pH 3.5 to 6 were monitored in sodium acetate buffer (100 mM) and pH 6 to 7.5 in 100 mM phosphate-citrate buffer. [file 2191-0855-1-14-S1.DOC]

## Additional files

Article title

**Study of enzymatic properties of phenol oxidase from nitrogen-fixing *Azotobacter chroococcum***

Journal name

**AMB Express**

Author names

**Susanne Herter*^1^, Marlen Schmidt^2^, Mark L. Thompson^2^, Annett Mikolasch^1^, Frieder Schauer^1^**

Affiliation

**^1^ Institute of Microbiology, Department of Applied Microbiology, University of Greifswald, Friedrich-Ludwig-Jahn-Str. 15a, 17489, Greifswald, Germany**

**^2^ Institute of Biochemistry, Department of Biotechnology & Enzyme Catalysis, University of Greifswald, Felix-Hausdorff Str. 4, 17489, Greifswald, Germany**

Email address of corresponding author*

[**susanne.herter@uni-greifswald.de**](mailto:susanne.herter@uni-greifswald.de)

## Additional file 1
