# Supplementary material for: Study of enzymatic properties of phenol oxidase from nitrogen-fixing Azotobacter chroococcum
Source: AMB Express. 2011 Jun 24;1:14. doi: 10.1186/2191-0855-1-14 (PMC3402154; doi:10.1186/2191-0855-1-14)
Supplement: Additional file 2 — Thermal stability of A. chroococcum PO comprised in crude extracts monitored with ABTS and 2,6-DMP. Thermal stability determined with ABTS (filled square) and 2,6-DMP (filled triangle), with samples pre-incubated for 30 minutes in NAc buffer (100 mM, pH 5) at temperatures ranging from 25-50°C. Error bars refer to standard deviation by means of four replicates. [file 2191-0855-1-14-S2.DOC]

## Additional file 2
